# Yale School of Public Health Symposium on tissue imaging mass spectrometry: illuminating phenotypic heterogeneity and drug disposition at the molecular level

**DOI:** 10.1186/s40246-018-0142-x

**Published:** 2018-02-27

**Authors:** Georgia Charkoftaki, Nicholas J. W. Rattray, Per E. Andrén, Richard M. Caprioli, Steve Castellino, Mark W. Duncan, Richard J. A. Goodwin, Kevin L. Schey, Sheerin K. Shahidi-Latham, Kirill A. Veselkov, Caroline H. Johnson, Vasilis Vasiliou

**Affiliations:** 1 Department of Environmental Health Sciences, Yale School of Public Health, Yale University, New Haven, USA; 2 Biomolecular Mass Spectrometry Imaging, National Resource for Mass Spectrometry Imaging, Department of Pharmaceutical Biosciences, Uppsala University, Uppsala, Sweden; 3 Departments of Biochemistry and the Mass Spectrometry Research Center, Vanderbilt University School of Medicine, Nashville, USA; 4 Department of Bio-Imaging, Platform Science and Technology, GSK, King of Prussia, USA; 5 Biodesix Inc., Boulder, USA; 6 Pathology, Drug Safety and Metabolism, IMED Biotech Unit, AstraZeneca, Cambridge, UK; 7 Departments of Biochemistry and Ophthalmology and Visual Sciences, Vanderbilt University School of Medicine, Nashville, USA; 8 Department of Drug Metabolism and Pharmacokinetics, Genentech, Inc., South San Francisco, USA; 9 Computational and Systems Medicine, Department of Surgery and Cancer, Faculty of Medicine, Imperial College London, London, UK; 10 Yale Cancer Center, Yale School of Medicine, Yale University, New Haven, USA; 11 Department of Ophthalmology and Visual Science, Yale School of Medicine, Yale University, New Haven, USA

## Introduction

‘A picture is worth a thousand words’ is an idiom from the English language (‘borrowed’ from on old Chinese proverb) that conveys the notion that a complex idea can be succinctly and fully described by a single image. Never has this expression been truer than in the clinical and pharmaceutical arenas. Enormous strides have been made by the scientific community in the evolving field of biomedical imaging with the aim of representing and/or quantifying aspects of disease and drug action by using tools such as radiography [1], MRI [2, 3] PET [4], and ultrasound [5]. Yet linking the phenotypical data generated by these systems to the genome is a challenging task. Identifying the link between the mechanism of disease or failed drug response to the genome of an individual is difficult, because central pieces of information are missing. However, imaging mass spectrometry (IMS) can overcome this issue. IMS aims to detect the molecular constituents of the tissue; these can then be correlated with genome-related characteristics, such as gene expression patterns and possible mutations, and ultimately provide a phenotypic molecular link to the complex disease biology. The big data technology of IMS can generate spatial information of thousands of metabolites and proteins from within a tissue, facilitating a deeper understanding of the connections between the genome, phenotypic characteristics and the biological response. It is a technology that has the potential to serve as a segue between gene expression and observed biological signal.

Image analysis has been a focus of mass spectrometry for more than 40 years since early studies using secondary ion mass spectrometry (SIMS) [6]. Among the several ionization techniques, matrix-assisted laser desorption ionization (MALDI) imaging mass spectrometry is the leader for analyzing molecular distributions within tissues [7]. MALDI IMS is capable of mapping biomolecules of interest at high spatial resolution (~ 1 μm), and high sensitivity. It can be employed to image a broad variety of molecular classes, from low-molecular-weight metabolites, lipids (> 1 kDa) and proteins [8, 9]. The unique ability of this technique to reveal these ionized molecular entities, while retaining the spatial information for multiple molecules in one measurement, makes histology-directed MALDI IMS a powerful tool for clinical applications and genome-based personalized medicine [10]. Furthermore, desorption electrospray ionization (DESI) [11] is an ionization technique that has the capability of direct solid surface sampling under open ambient conditions. DESI has the advantages of ambient ionization methods and combined with MALDI, hundreds-to-thousands of molecules can be evaluated simultaneously and their spatial distribution can be visualized from within the same tissue section (Table 1). Consequently, the molecular changes in a tissue can be accurately studied, correlated to images and cellular features generated by traditional histology, and the pathogenic mechanisms of a certain disease can be visualized and identified, leading to the potential discovery of new biomarkers [8].

**Table 1:** Imaging mass spectrometry ionization techniques: application, advantages, and disadvantages [8, 9, 11].

Application	Ionization	Advantages	Disadvantages
Tissue molecular imaging	MALDI	• Label-free analysis • High-sensitivity • Use over a broad mass range • High-spatial resolution (≤ 1 μm) • Application on both formalin-fixed paraffin-embedded (FFPE) tissue microarrays and on fresh tissue samples.	• Low throughput • Sample preparation can lead to spatial dislocation or chemical modifications • Matrix dependent analysis
	DESI	• Label-free analysis • Ambient ionization method • Direct solid surface sampling • Multiple charged ions • Minimal sample preparation • Soft ionization method • Generally less costly upon comparison to MALDI	• Imaging in the low-mass region—limited use for proteins • Poorer spatial resolution compared to MALDI • Solvent dependent

The pharmaceutical industry has taken advantage of the development of IMS to enable an array of high-throughput screening modalities for pharmaceutical assessments [12]. IMS can provide reliable, label-free qualitative and quantitative distribution information for a drug of interest and its subsequent biotransformed metabolites [13]. This information can be used to determine and understand the pharmacokinetic (PK) properties of a drug, its penetration into tissue, and to assess drug efficacy and potential toxicity [14]. This makes IMS a powerful, yet cost-effective technology because distribution studies can be performed earlier in the drug discovery process without any requirement for radiolabeled standards. Of critical importance, IMS separately maps and differentiates drug from its metabolites, rather than tracking just a radiolabeled parent drug [14–16].

At this 1-day symposium, the Department of Environmental Health Sciences (DEHS) at the Yale School of Public Health brought together leaders in IMS to discuss recent developments, limitations, and future needs, and to increase awareness of this growing and important field. During his opening remarks, Dr. Vasiliou highlighted the potential of IMS to define the molecular basis of diseases, to provide insights into mechanisms, and to integrate tissue morphology at the molecular level. Thereafter, talks by an impressive assembly of thought-leaders in IMS illustrated the potential of unbiased tissue imaging to deliver a new level of understanding of pathophysiological processes at the molecular level. The symposium concluded with a round-table discussion, chaired by Dr. Mark Duncan, on some of the more practical issues in IMS such as current bottlenecks and future opportunities. The presentations and discussions at the symposium underscored the great potential of IMS. With the intention of bringing IMS to the larger scientific community, the DEHS has committed significant resources to the acquisition of equipment and expertise that will allow the further development and application of IMS techniques.

## MALDI imaging mass spectrometry (IMS): recent technological advances

The first talk of the symposium was given by a pioneer of the field, Dr. Richard Caprioli, Professor of Biochemistry and Director of the Mass Spectrometry Research Center at Vanderbilt University. His opening remarks gave a historical overview of MALDI IMS technology and emphasized its advantages. Dr. Caprioli explained how MALDI IMS employs desorption of molecules by direct laser irradiation to map the location of specific molecules from fresh frozen or formalin-fixed tissue sections without the need to target specific reagents, such as antibodies [17]. Dr. Caprioli championed the major benefits of the histology-directed approach (that has been developed by his group) over conventional staining and microscopic methods. This technology is an addition to the histologist’s toolbox, not a replacement. By integrating microscopy with MALDI IMS, this application is almost limitless and could be used in a variety of biologically and medically relevant research projects. Dr. Caprioli highlighted studies in diabetic nephropathy involving both proteins and lipids and the differentiation of benign skin lesions from melanomas [17, 18]. In addition, Dr. Caprioli’s group has applied IMS to drug targeting and metabolic studies in specific organs and in intact whole animal sections following drug administration [19]. Recent technological advances were also described for sample preparation to improve metabolite extraction and instrument performance to achieve images at high spatial resolution (1–10 μm) and at high speeds so that a typical sample tissue, once prepared, can be imaged in minutes [20]. Instrumentation used in these studies included both MALDI fourier transform ion cyclotron resonance (FTICR) and MALDI time-of-flight (TOF) mass spectrometers. Applications utilize tandem mass spectrometry (MS/MS), ultra-high mass resolution, and ion accumulation devices for IMS studies. Finally, new biocomputational approaches were discussed that are required to handle the high-data dimensionality of IMS, and also ‘image fusion’ for predictive integration of mass spectrometry (MS) images with microscopy and other imaging modalities [21].

Dr. Kevin Schey, Professor of Biochemistry, Ophthalmology and Visual Sciences, at Vanderbilt University, discussed the application of IMS to study a range of molecular classes, such as proteins, lipids, and metabolites in ocular tissues. Ocular tissues provide an ideal medium to demonstrate the utility of the technique where morphological features are on the scale of single cells. For example, molecular profiles can be produced in retina pigment epithelium. Moreover, a range of diseases affect the various ocular tissues, including glaucoma, age-related macular degeneration, cataract, and corneal cataract. Dr. Schey illustrated how IMS is being actively applied to derive mechanistic information that enhances understanding about the molecular underpinnings of disease in these tissues as well as aging mechanisms. IMS data from optic nerve, retina, lens, and cornea were presented with special attention to diseases affecting these tissues [22]. Data from both animal models of disease and human tissues were discussed, as well as key methodological details for successful imaging ocular tissues [23–25]. Dr. Schey’s work, in collaboration with Dr. Vasiliou, on a corneal haze phenotype in *Aldh3a1-null* mice presented the first genetic animal model of cellular-induced corneal haze due to the loss of a corneal crystallin [24]. This work clearly showed how IMS can provide deeper understanding for the genome, linking the disease phenotype with genetic changes.

The broad range of IMS applications was reinforced even more by Dr. Andr*é*n, Professor at Uppsala University, who showed novel ways to interrogate the actions of neurotransmitters, their precursors and metabolites, in the brain chemical network and neuronal signal transmission [26]. Changes in neurotransmitter concentrations are associated with numerous normal neuronal processes, such as sleep and aging, and in several disease states, including neurological disorders (e.g., Parkinson’s and Alzheimer’s disease), depression, and drug addiction. Dr. Andr*é*n uses knowledge about the relative abundance and spatial distribution of neurotransmitters in the brain to provide insights into these complex neurological processes and disorders. At present, researchers rely on indirect histochemical, immunohistochemical, and ligand-based assays to detect small-molecule transmitter substances or on tissue homogenates analyzed by high-performance liquid chromatography analysis. Current neuroimaging techniques have very limited capacities to directly identify and quantify neurotransmitters from brain sections. MALDI IMS can perform analyses directly on the surface of a tissue section, establishing itself as a powerful in situ visualization tool for measuring abundance and spatial distribution of endogenous and pharmaceutical compounds, lipids, peptides, and small proteins. A novel reactive MALDI matrix, recently developed by Dr. Andr*é*n’s group, selectively targets the primary amine group on neurotransmitters, metabolites, and neuroactive substances while also functioning as a matrix to enable ionization [27]. However, the limitation of using such a reactive matrix to study the full molecular pathways of, for example, dopaminergic or serotonergic biosynthesis and metabolism is its limitation to target all downstream dopamine metabolites derived from monoamine oxidase (MAO) or catechol-O-methyltransferase (COMT) enzymes. The majority of small molecule neurotransmitters, such as catecholamines, amino acids, and trace amines, possess phenolic hydroxyl and/or primary or secondary amines which are strong nucleophilic groups. Dr. Andr*é*n’s laboratory has therefore developed a new reactive matrix that can selectively target and charge-tag both phenolic and primary amine groups, thus enabling MALDI IMS of both MAO and COMT downstream metabolites, focusing on a nucleophilic aromatic substitution reaction with such functional groups. Using this new reactive matrix, they were able to detect and map the localization of most of the neurotransmitters and metabolites involved in the dopaminergic and serotonergic network in a single brain tissue section. This work showed a novel methodology that assists with metabolite identification through the selectivity of the reaction. The sensitivity and specificity of this imaging approach to neurochemicals has great potential for many diverse applications in neuroscience, pharmacology, drug discovery, neurochemistry, and medicine.

## Visualizing drug disposition in tissue

A major focus of the symposium was the application of MALDI IMS to map the distribution of a variety of therapeutic molecules across a tissue section of interest and to assess their biological impact.

Current president of the Imaging Mass Spectrometry Society and director of US Imaging MS, at GlaxoSmithKline (GSK), Dr. Castellino, discussed how MALDI IMS technology has taken their research beyond “plasma-centric” studies and allowed for direct mapping of molecular changes in tissue associated with drug pharmacology, disposition, and disease pathogenesis. Delivering safe and efficacious drugs is tied to the ability to understand complex mechanistic relationships between molecular initiation events of pharmacologically active compounds and the cascade of subsequent biological consequences. Because the delivery of drugs to their intended target, and avoidance of unintended targets, is a critical first step, IMS can directly guide improvements and innovation in delivery strategies by mapping the target tissue selectivity [28]. Furthermore, tissue correlations can be directly made to plasma PK or lead to improved pharmacodynamic understanding. Dr. Castellino’s group has explored the use of MALDI IMS to investigate the distribution of drugs and their metabolites, as well as endogenous compounds, in a wide variety of target tissues in support of numerous therapeutic areas and in all drug discovery and development stages [29, 30]. The IMS methodology allows for the co-registration of drug analytes in tissue distributions with histology images, thereby integrating chemical structures with tissue morphology. Furthermore, this imaging modality offers the potential to further our mechanistic understanding of drug disposition, disease progression, and pharmacology (including toxicology) by providing snap shots of temporal and causal changes [31]. Dr. Castellino continued, that while MALDI IMS is primarily being employed to determine the tissue distribution of drugs and their metabolites, it has become evident that more detailed understanding of biological systems can be gained by including the changes in endogenous compound distribution as a function of disease and pharmacology. Closing his presentation, Dr. Castellino discussed the importance of suitable software tools and improved data handling methods needing to be developed alongside analytical progress in order for the full potential of MALDI IMS to be realized.

Dr. Richard Goodwin, a principal scientist for Drug Safety and Metabolism at AstraZeneca (AZ) and head of mass spectrometry imaging, presented the challenges faced for drug discovery and development; it is a lengthy, high risk, and competitive business that can take a decade to progress; moreover, billions of dollars are required to move a new medicine to market. Dr. Goodwin discussed how IMS could help mitigate some of the primary reasons for drug attrition, specifically around lack of efficacy and toxicological or clinical safety risk. IMS is now demonstrating impact on drug discovery programs and helping reduce later stage compound attrition. It provides insights into the biodistribution of compounds, while simultaneously generating data on pharmacodynamic biomarkers. Dr. Goodwin presented data from AZ that showed how the use of a range of multimodal imaging techniques improves understanding about compound efficacy, safety, and targeted drug delivery [32, 33]. Investigating histopathological-targeted drug-induced toxicity is now readily achieved using high-spatial resolution and high-mass resolution IMS. Dr. Goodwin outlined how a Cancer Research UK Grand Challenge consortium are seeking to use multimodal IMS to offer new insights into tumor metabolism and to help develop new, more effective medicines and therapy combinations. The $20 million project led by Professor Bunch at the National Physical Laboratory UK (in collaboration with world leading oncology biologists, IMS technologists, and AZ) will utilize data similar to that shared at the symposium. IMS can help identify metabolite changes consistent with the biomarker changes in the tumor and show changes in metabolites as PD biomarkers, hence providing valuable new insights into the pathway and drug combinations. The next hurdle is how to effectively mine multimodal imaging data. Recent strategies on data processing and visualization as well as data mining algorithms were outlined [34]. In his closing remarks, Dr. Goodwin highlighted the challenges and opportunities arising from the significant quantities of molecular imaging data generated, from a cellular to patient level.

Dr. Sheerin Shahidi-Latham, Head of Metabolomics and Imaging MS, Department of Drug Metabolism and Pharmacokinetics at Genentech Inc., also discussed the advantages of the applied use of MALDI IMS. In the pharmaceutical industry, obtaining information about the absorption, distribution, metabolism, and elimination (ADME) of a new chemical entity via a PK study in a preclinical animal model is often the first step towards understanding the in vivo properties of a drug-like molecule in humans. Traditionally, much of this ADME work has been supported by liquid-chromatography coupled to mass spectrometry. In the case of tissue distribution, the organs are excised and homogenized in order to accommodate this analytical workflow, thereby effectively eliminating any spatial information. Dr. Shahidi-Latham emphasized that MALDI IMS has gained prominence since it provides a robust, label-free detection of drug and metabolites while preserving spatial localization within tissue sections of interest. Additionally, the use of high-resolution mass spectrometers has provided the opportunity for simultaneous detection of subsequent pharmacodynamic (PD) responses within a single image acquisition. Dr. Shahidi-Latham discussed how the ability to assess PK/PD relationships in a label-free, in situ context has proven invaluable to the early lead optimization efforts that take place in the drug discovery setting. Similarly, uncovering the perpetrator of adverse effects often associated with histopathological assessments in the preclinical development phase has also improved their understanding about the mechanisms of toxicity and can provide useful information for the redesign of a back-up molecule. The presentation provided a synopsis of the advantages of IMS, as well as the technical challenges and opportunities in the context of the pharmaceutical industry. Dr. Shahidi-Latham presented data from her work at Genentech, which included MALDI IMS of dosed tissues in support of drug efficacy, PK/PD, and effective delivery evaluations, and highlighted the simultaneous detection of drug, metabolites, and endogenous components attainable from a single imaging run [35–39]. Moreover, examples demonstrating the utility of imaging MALDI IMS for toxicity screening were presented. Its complementary use with autoradiography analyses were discussed, including ocular drug distribution and whole-body drug disposition studies [15].

## Bioinformatics platform for large-scale mass spectrometry imaging data

The constant drive towards integrating complex and large-scale datasets has generated the need to develop new tools to process this information [40]. Dr. Kirill Veselkov, Lecturer at Imperial College London, discussed how managing, analyzing, and interpreting these data are a challenge and a major barrier to their clinical translation. IMS augments digital pathologic analysis with highly robust big data on cellular metabolic and proteomic molecular content, generating a staggering amount of unrefined data (tens to hundreds of gigabytes of data per tissue section). Existing data analysis solutions for IMS rely on a set of heterogeneous bioinformatics packages that are not scalable for the reproducible processing of large-scale (hundreds to thousands) biological sample sets. In this talk, Dr. Veselkov presented a computational platform (pyBASIS) capable of optimized and scalable processing of IMS data for improved information recovery and comparative analysis across tissue specimens using machine learning and related pattern recognition approaches. The proposed solution also provides a means of seamlessly integrating experimental laboratory data with downstream bioinformatics interpretation and analyses, resulting in a truly high-throughput system for translational IMS.

The symposium concluded with a round-table discussion, chaired by Dr. Mark Duncan, where the attendees discussed the practical challenges and future directions of IMS. The attendees not only agreed on the potential of IMS to provide previously inaccessible insights into molecular events at the tissue level, but also highlighted the cost and complexity of both the science and the technology that underpins these studies. Rather than a routine core service, it was agreed that designing meaningful studies, performing exacting sample handling, generating and interpreting complex data, and maintaining high-end instrumentation requires a substantial, highly collaborative interaction between all stakeholders.

## Abbreviations

ADME: Absorption, distribution, metabolism, and elimination
COMT: Catechol-O-methyltransferase
DESI: Desorption electrospray ionization
IMS: Imaging mass spectrometry
MALDI: Matrix-assisted laser desorption ionization
MAO: Monoamine oxidase
MS: Mass spectrometry
PD: Pharmacodynamic
PK: Pharmacokinetic
